# Acute effects of ethanol on GABA_A_ and glycine currents in the lateral habenula neurons of young rats

**DOI:** 10.13055/ojns_3_1_5.130821

**Published:** 2013-08-21

**Authors:** Zijing Xie, Guohui Li, Jiang-Hong Ye

**Affiliations:** a Department of Anesthesiology, Pharmacology and Physiology, University of Medicine and Dentistry of New Jersey, New Jersey Medical School, Newark, New Jersey; b Department of Neurology Dong-Zhi-Men Hospital, Beijing University of Chinese Medicine. Key laboratory for internal Chinese Medicine of Ministry of Education, China

## Abstract

Compelling evidence has shown a pivotal role of dopaminergic function in drug addiction. Recently, the lateral habenula (LHb) has attracted a great deal of attention as another target for abused drugs in the brain because its role in regulating dopaminergic system, among others. GABA and glycine are major inhibitory neurotransmitters. Their corresponding receptors are key targets of ethanol. The properties of these receptors in LHb neurons and their responses to ethanol in particular however, remain unknown. Using the patch clamp techniques, we examined the effects of ethanol on the chloride currents elicited by GABA and glycine in LHb neurons acutely dissociated from 10-20 day-old Sprague–Dawley rats. We show that GABA concentration-dependently elicited a bicuculline sensitive inward current in 96% (130/140) of the neurons tested. Ethanol (43.2 mM) suppressed current elicited by a wide range of concentrations (1-300 μM) of GABA in 74% (35/47) cells tested. Ethanol suppression is dependent on its concentrations but not on membrane potentials of the neurons. Moreover, glycine concentration-dependently elicited an inward current in 94% (112/120) of the neurons tested. Both strychnine and picrotoxin concentration dependently suppressed glycine current with IC_50_ of 220 nM and 813 μM, respectively. Ethanol (43.2 mM) potentiated current elicited by unsaturated but not saturated concentrations of glycine. Thus, the LHb neurons of young rats contain both functional GABA_A_ and glycine receptors which are sensitive to ethanol at pharmacologically relevant concentrations. These effects of ethanol might be important in the control of the activity and output of LHb neurons.

## INTRODUCTION

Alcohol is among the most frequently abused drugs in our society. It is generally accepted that ligand gated ionic channels (LGICs) are the major targets of ethanol. Of these LGICs, γ-aminobutyric acid type A (GABA_A_) and glycine receptors appear to occupy a central role in mediating the effects of ethanol in the CNS [1]. They both are chloride channels and the primary inhibitory neurotransmitters in the mammalian CNS. Their activation tends to decrease neuronal excitability. Many previous studies have shown that ethanol enhances GABA_A_ currents in various preparations [2], including hippocampal and cortical neurons of mice [3], dorsal root ganglion neurons [4], retinal bipolar cells and ganglion cells [5] and locus coeruleus neurons of rats [6].

Glycine receptors (GlyRs), like GABA_A_Rs, are chloride channels, represent the primary fast inhibitory mechanisms in central nervous system. GlyRs are best known in the spinal cord and the lower brainstem. However, GlyRs are widely distributed throughout the mammalian CNS [7]. GlyR contains four α-subunits (1–4) and one β-subunit. Previous studies have indicated that in naïve neurons, functional GlyRs are comprised of α-homomers and α–β heteromers with a subunit stoichiometry of 2α3β [8, 9] and that the subunit composition and their assembly change with development [9b]. In contrast to the numerous studies on GABARs, studies of the effects of ethanol on GlyRs are fewer and more limited in scope. Engblom and Akerman [10] reported that ethanol potentiates glycine-activated Cl^−^ uptake into synaptoneurosomes of whole-rat brain. In addition, central depressant effects of ethanol were shown to be enhanced by glycine and the glycine precursor serine [11]; the specific antagonist strychnine blocked this action, indicating that glycine enhances ethanol effects via strychnine-sensitive GlyRs [12]. Ethanol’s positive modulatory effect on recombinant GlyRs was shown to be determined by a single amino acid in the subunit of the strychnine-sensitive GlyR [13]. Electrophysiological studies are supportive, revealing a positive modulation of glycine current by ethanol in cultured neurons from chicks [14], mice [15], rats [16], Xenopus oocytes and mammalian cell lines expressing homomeric GlyRs [13a, 17]. However, data from LHb neurons are lacking. Here, using patch clamp techniques, we show that pharmacologically relevant concentrations of ethanol (10.8-43.8 mM) reduces response of GABA but increases response of glycine in neurons acutely dissociated from the LHb.

## MATERIALS AND METHODOLOGY

### Isolation of Neurons

The care and use of animals and the experimental protocol were approved by the Institutional Animal Care and Use Committee of the University of Medicine and Dentistry of New Jersey. The brain slices were prepared as described previously [7b]. In brief, 10-20 day-old rats of both sexes were anesthetized and then killed by decapitation, and the brain was quickly excised and coronally sliced (300 μm) with a VF-200 Slicer (Precisionary Instruments, Greenville, NC). This was done in ice-cold modified glycerol-based artificial cerebrospinal fluid (aCSF) saturated with 95%O2/5% CO_2_ (carbogen) containing (in mM): 252 glycerol, 2.5 KCl, 1.2 NaH_2_PO_4_, 1.2 MgCl_2_, 2.4 CaCl_2_, 26 NaHCO_3_, and 11 glucose [18]. Slices were then kept in carbogen-saturated regular aCSF at room temperature (22–24 °C) for at least 1 h before use. The regular aCSF has almost the same composition as glycerol-based aCSF, the exception being that the 252 mM glycerol was replaced with 126 mM NaCl.

The standard external solution in which the currents were recorded containing (mM) 140 NaCl, 5 KCl, 1 MgCl_2_,2 CaCl_2_,10 glucose, and 10 HEPES. The pH was adjusted to 7.4 with Tris base and the osmolarity to 320 mM with sucrose. To obtain dissociated neurons, slices containing the LHb were first incubated in oxygenated standard solution containing 4 mg/ml papain at 31°C for 30 min. The LHb region was cut out under an inverted microscope and single cells were dissociated by trituration using two fire-polished glass pipettes with gradually narrower diameters. The cells settled to the bottom of the culture dish within 20 min and were ready for electrophysiological recordings.

### Electrophysiological measurements

Whole-cell configurations were used to record currents with an Axopatch 1D amplifier (Molecular Devices Inc., Foster city, CA), via a Digidata 1322A analog-to-digital converter (Molecular Devices), and pCLAMP 9.2 software (Molecular Devices). Data were filtered at 1 kHz and sampled at 5 kHz. The patch electrodes had a resistance of 3–5 MΩ when filled with pipette solution containing (in mM): 120 CsCl, 21 TEA, 4 MgCl_2_, 11 EGTA, 10 CaCl_2_, 10 HEPES, and 2 Mg-ATP. The pH was adjusted to 7.2 with Tris base, and the osmolality was adjusted to 280–300 mosM with sucrose. Since the GABA_A_ response recorded by the conventional patch-clamp technique decreases time-dependently, 5 (instead of 2) mM MgATP was added to the pipette solution to minimize the run-down. Electrophysiological recordings were performed at room temperature (22–24 °C).

### Chemicals and application

Most of the chemicals including GABA, glycine, bicuculline, strychnine, picrotoxin and papain were purchased from Sigma-Aldrich Inc. (St. Louis, MO). Ethanol was obtained from Pharmco (Brookfield, CT). All solutions were prepared on the day of the experiment. Chemicals were applied to dissociated neurons with a Y-tube. This exchanged the extracellular solution surrounding the neurons within 40 ms [19].

### Data analysis

The percentage of change of a current (GABA or glycine) by an agent was calculated by using the formula (B / ((A+C)/2) × 100), where (A) is the amplitude of current during baseline conditions, (B) during agent application, and (C) after washout of the agent. Concentration-response data were analyzed with a nonlinear curve-fitting program (Sigma Plot, Jandel Scientific). Data were statistically compared using One way ANOVA or Student’s t-test where appropriate and at a significance level of P < 0.05, or as otherwise indicated. For all experiments, average values are expressed as mean ± SEM. To obtain a concentration-response relationship for GABA_A_Rs or GlyRs, all neurons were exposed to three or four concentrations of GABA/glycine, in the range of 0.01–1 mM. For each concentration, four to six responses from a given neuron were normalized to the peak current evoked by 3 μM GABA or 100 μM glycine. The normalized values from three to five neurons at each concentration of GABA/glycine were averaged. Using a Simplex algorithm (Sigma plot, Jandel Scientific), these averages were then fitted to the Hill equation: I = I_max_/[1+EC_50_/C^*n*^], where I, I_max_, C, EC_50_ and *n* are I_GABA_(I_Gly_), maximal I_GABA_(I_Gly_), concentration of GABA(glycine), the concentration for 50% of maximum response, and the Hill coefficient, respectively.

## RESULTS AND OBSERVATIONS

### Response of LHb cells to GABA

**Figure 1:**
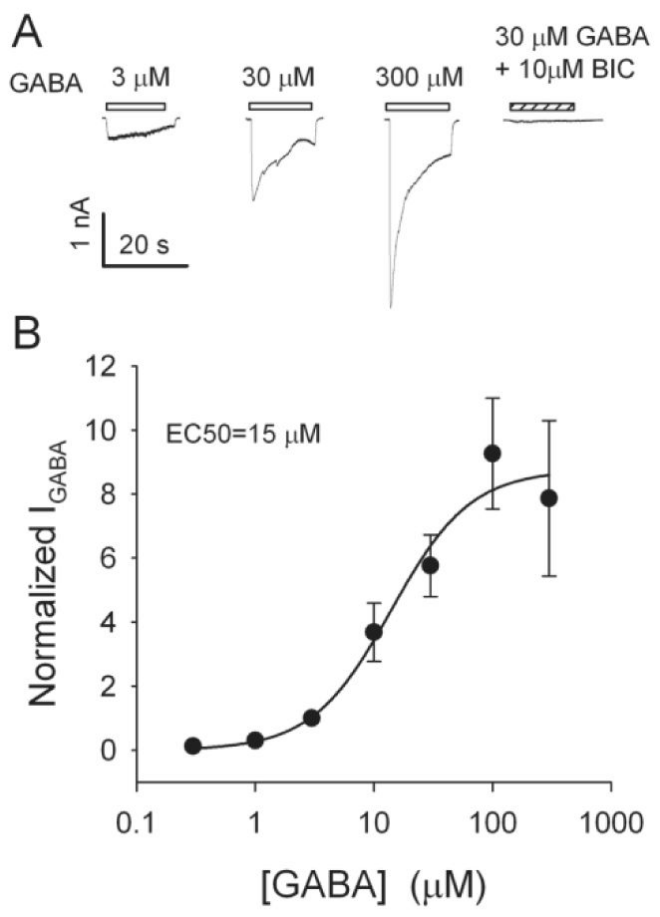
GABA currents GABA-induced currents in LHb neurons. A: Exemplar current traces recorded from an LHb neuron in response to GABA at the indicated concentrations. For this and all figures, currents were obtained at a V_H_ of −60 mV. This current was blocked by 10 μM bicuculline (BIC, right panel). B: Concentration-response relationship of GABA. All responses were normalized to the peak current amplitude induced by 3 μM GABA. Each data point is the mean (±S.E.M.) from 5-10 neurons. Solid lines are the fit of the Hill equation described in the method section to the data. The EC_50_ and n were 15 μM and 0.89, respectively.

Approximately 96% (134/140) of the LHb neurons examined produced an inward current (I_GABA_) in response to GABA at a V_H_ of −60 mV. In response to a threshold concentration (between 0.1 to 1 μM), the current was slow in onset and did not decay during the application of the agonist. However, with the increase of GABA concentrations, there was a progressive increase in the peak amplitude and rate of onset, as well as the rate of decay. This current was abolished by 10 μM bicuculline and further confirmed that it was mediated by GABA_A_Rs (Fig. 1A). The concentration dependence of the peak current was well fitted by the Hill equation (r2 = 0.89), giving an EC_50_ of 15 μM (Fig. 1B).

### Ethanol inhibits I_GABA_

No discernible currents were observed when ethanol was applied alone at concentrations of ≤ 43.2 mM. However, when co-applied with GABA, 43.2 mM ethanol inhibited I_GABA_ of 74% (35/47) of the neurons tested.

**Figure 2:**
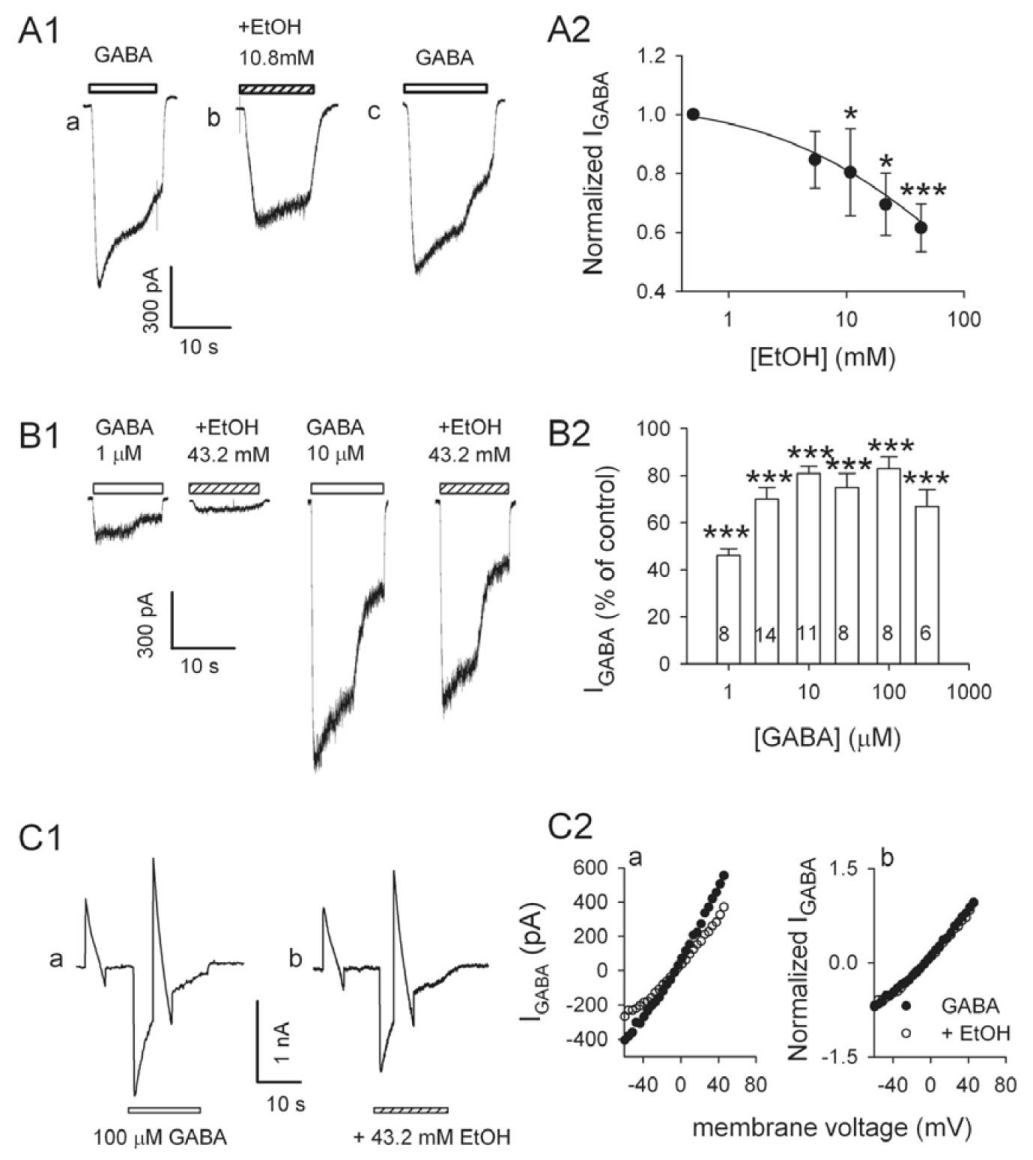
Ethanol suppresses GABA currents Ethanol depresses I_GABA_ of LHb neurons. A: Ethanol (EtOH) reversibly and concentration-dependently suppressed I_GABA_. A1, Typical current traces from an LHb neuron elicited by 10 μM GABA alone (a and c) or together with 10.8 mM ethanol (b). A2: Ethanol concentration dependently depresses I_GABA_. Data were mean (±SEM) of 6 to 8 neurons at each concentration. Data were normalized to the peak current induced by 10 μM GABA alone. For estimation of the IC_50_ and n of the concentration–response curve, the following form of the Logistic equation was fit to the data, I/I_GABA_ =1/(1+(C+(K_d_)^n^). Where I is the current with ethanol, I_GABA_ is the control current, and C is the concentration of ethanol. IC_50_ and n were 94 mM and 0.97, respectively (*P<0.05, ***P<0.001). B: Ethanol depression of current induced by a range of concentrations of GABA. B1, typical current traces induced by 1 and 10 μM GABA in the absence and presence of 43.2 mM ethanol. B2: Ethanol (43.2 mM) suppressed current induced by 1-300 μM GABA. Data (mean ± S.E.M) were normalized to the peak amplitude induced by GABA alone in each concentration. Cell numbers are indicated. C: Ethanol-induced depression of I_GABA_ is independent of membrane voltage. GABA current –voltage relation was studied with pairs of voltage ramps (from +60 mV to −60 mV) applied at a rate of 1 mV/10 ms, as illustrated in C1. Drugs were applied to the cell and cover the second ramp in each pair. Traces obtained from the first ramp served as background. Subtracting the trace obtained in the first ramp from that in the second ramp produced the I-V curve. C1, typical I_GABA_ recorded from a neuron exposed to 100 μM GABA alone (a) and in the presence of 43.2 mM ethanol (b). C2: I-V curves derived from C1 shows that ethanol suppressed I_GABA_ at all potentials without changing the apparent reversal potential of this current. Similar data were obtained from three other cells. C2(b): to determine the voltage dependence, current recorded in control and in the presence of ethanol were first normalized to the value obtained at −60 mV. Normalized I-V relations from the same experiment as C2(a) shows ethanol suppression is not voltage dependent.

We first examined the effects of ethanol on the currents induced by 10 μM GABA. This concentration of GABA was chosen because it was close to the EC_50_ value of the LHb neurons. Fig.2A shows typical current traces activated by 10 μM GABA alone (A, a) and in the presence of 10.8 mM ethanol (A, b); the currents recovered to the control level after washout of ethanol (A, d). At concentrations between 10.8 and 43.2 mM, ethanol reduced I_GABA_ in a concentration-dependent manner (Fig.2A2). The means of normalized ethanol inhibition is plotted as a function of the ethanol concentrations. On average, 10.8 and 43.2 mM ethanol decreased the peak current induced by 10 μM GABA to 80 ± 14 % (n = 9) and 61 ± 8 % (n =10) of control, respectively. The analysis of the data reveals that ethanol suppressed I_GABA_ induced by 10 μM GABA with an IC_50_ of 94.1 ± 29.1 mM and r2 of 0.97.

We next examined the concentration-response relationships for GABA in the presence of 43.2 mM ethanol (Fig.2B). On average, 43.2 mM ethanol significantly decreased the peak currents activated by 1, 3, 10, 30, 100, 300 μM GABA to 46±3% (n=8), 70±5% (n=14), 81±3% (n=11), 75±6% (n=8), 83±5% (n=8), and 67±7% (n=6) of control, respectively (P<0.001). Note that although ethanol significantly suppressed the I_GABA_ induced by 1-300 μM GABA, the suppression for 1 μM GABA is significantly greater than that for 300 μM GABA (P=0.01, unpaired *t* test).

**Figure 3:**
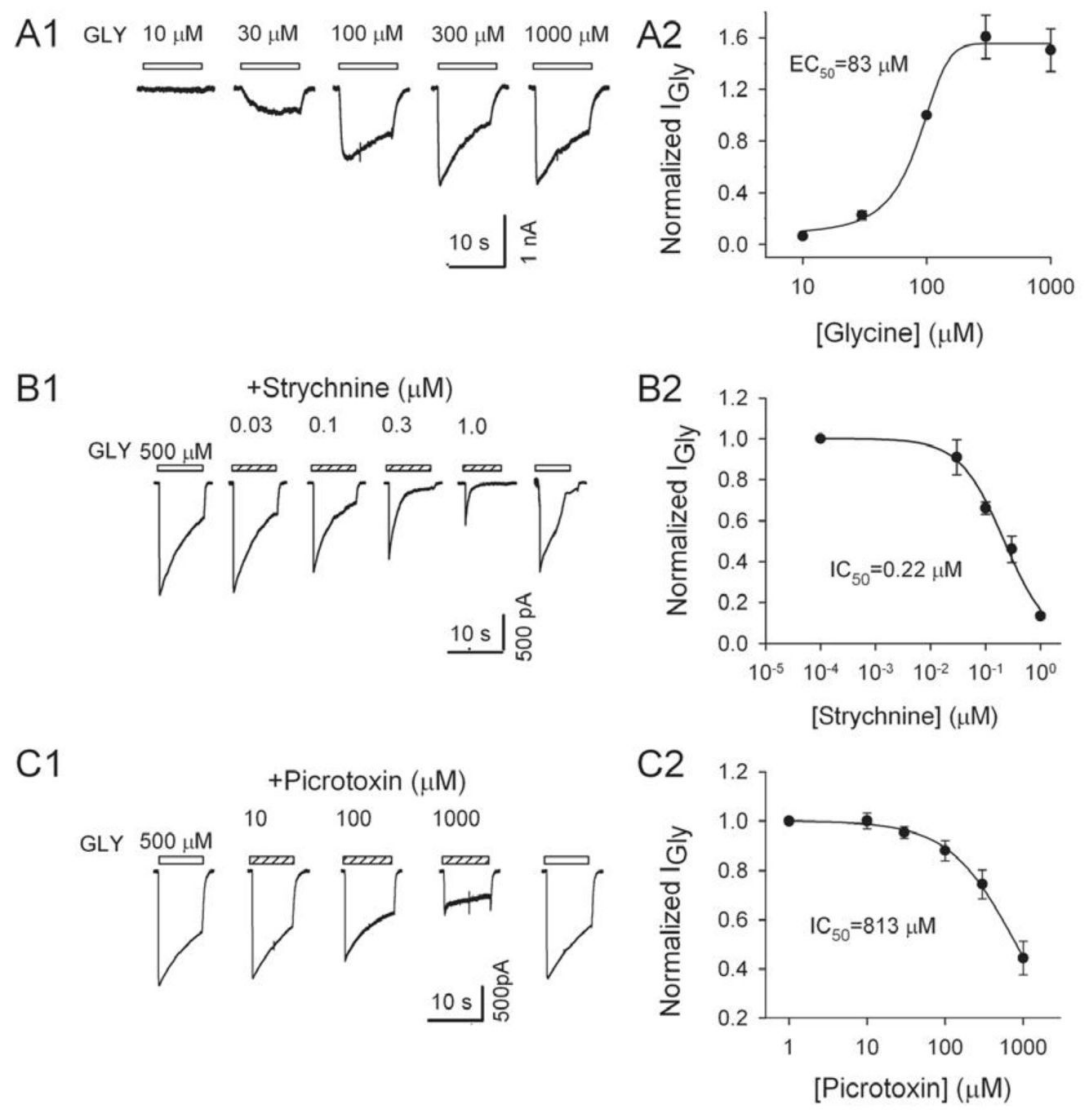
Glycine currents Glycine-induced currents (I_Gly_) in LHb neurons. A, Typical I_Gly_ traces of LHb neurons (A1). (A2) Concentration-response curves of I_Gly_. All points were normalized to the peak response elicited by 100 μM glycine. Each point is the mean of 14 cells and the vertical bars show ± S.E.M. The EC_50_ (83.3 μM) and n (0.99) were estimated using the Hill equation described in Method. B: Strychnine dose-dependently suppresses I_Gly_. B1, Typical current traces of an LHb neuron in response to 500 μM glycine in the absence and presence of strychnine at the concentrations indicated. B2, concentration–response relationship of strychnine blockage of I_Gly_. After normalizing the peak current in the presence of strychnine to the control value, the mean ± S.E.M. was calculated and plotted as a function of strychnine concentrations. Each point represents the mean of eight cells and the vertical bars show ± S.E.M. The IC_50_ (0.22 μM) and n (0.99) were estimated using the Logistic equation described in Fig. 2A. C, Picrotoxin suppression of I_Gly_. C1, Typical current traces in response to 500 μM glycine in the absence and presence of picrotoxin. C2, concentration–response relation of picrotoxin blockage of I_Gly_. After normalizing the peak I_Gly_ in the presence of picrotoxin to the control value, the mean ± S.E.M. was calculated and plotted as a function of picrotoxin concentrations. Each point represents the mean of three to six cells. The IC_50_ (813 μM) and the n (0.7) of the concentration-response curve were estimated using the Logistic equation described in Fig. 2A.

We then examined the current-voltage (I-V) relations of I_GABA_ obtained in the absence and presence of ethanol with a “ramp” protocol (fig. 3C1). The resulting I-V curves reveal that the suppressant effect of ethanol on I_GABA_ did not depend on the voltage, because the suppressant effect was the same at membrane potentials ranging from −100 to +60 mV (Fig.2C2). Furthermore, in the presence of ethanol, the GABA-activated channel remained selectively permeable to Cl^−^ since the reversal potential of I_GABA_ remained close to the calculated Nernst potential for Cl^−^, which is 0 mV in our experimental conditions.

In a small number of cells tested, 43.2 mM ethanol significantly enhanced current elicited by 1 and 3 μM of GABA to 121.9% ± 10.5%, (P=0.044, *n*=3), and 151.1% ± 42.0%, (P=0.026, *n*=7), respectively (data not illustrated).

### Responses of LHb cells to glycine

In 94% (112/120) of LHb neurons examined at a V_H_ of −60 mV application of glycine induced an inward current (I_Gly_) (Fig.3A1). I_Gly_ increased in amplitude sigmoidally with the concentration of glycine. The concentration dependence of the peak current was well fitted by a logistic equation (r^2^ = 0.99), giving EC_50_ of 83.3 (± 4.7) μM (mean ± SEM, Fig. 3A2).

### Effects of strychnine on I_Gly_

The plant alkaloid, strychnine, is a selective antagonist for GlyRs [20]. The α subunits of GlyRs carry the binding site for strychnine. To characterize the pharmacological properties of the GlyRs of LHb neurons, we tested the effects of strychnine on I_Gly_. The relationship between the peak amplitude of I_Gly_ (normalized to the peak amplitude of the control I_Gly_ induced by 500 μM glycine) and the concentrations of strychnine is illustrated in Fig.3B. Strychnine (0.03–1.0 μM) decreased I_Gly_ in a concentration dependent manner, which was well fitted by a logistic equation (r2 = 0.99), giving an IC_50_ of 0.22 (± 0.06) μM (mean ± SEM).

### Effects of picrotoxin on I_Gly_

The GABA_A_ antagonist picrotoxin is a useful tool in differentiating between homomeric and heteromeric GlyRs. Previous studies have shown that low concentration of picrotoxin suppresses the function of α homomeric GlyRs, but affects the function of α+β heteromeric receptors less [7c,^21^]. In order to obtain further information for the subunit structure of native GlyRs in the LHb neurons, we tested the effect of picrotoxin on I_Gly_. Picrotoxin concentration dependently reduced I_Gly_. The relationship between the peak amplitude of I_Gly_ (normalized to the peak amplitude of the control I_Gly_ induced by 500 μM glycine) and the concentrations of picrotoxin is illustrated in Fig.3C. Inhibition curve was fit to these data and the results yielded a picrotoxin IC_50_ of 813.3 ± 40.4 μM (mean ± SEM).

### Ethanol potentiates I_Gly_

**Figure 4:**
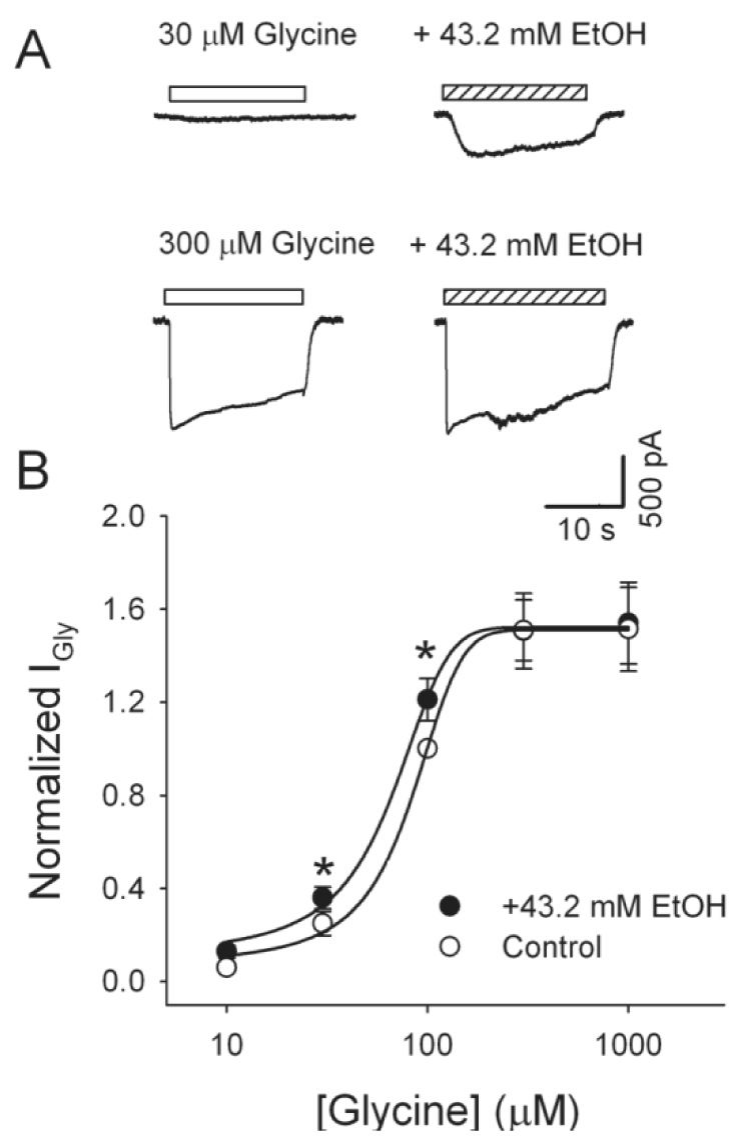
Ethanol enhances glycine currents Ethanol potentiates I_Gly_ of LHb neurons. A, Exemplar current traces from an LHb neuron elicited by glycine (30, and 300 μM) in the absence and presence of 43.2 mM ethanol. B: Ethanol-induced potentiation of I_Gly_ depends on the concentrations of glycine. Data were mean (±S.E.M.) of 6 to 8 neurons at each concentration. Data were normalized to the peak current induced by 100 μM glycine alone. The smooth curves were fit the data to the Hill equation described in Method. (*P<0.05).

To test the effect of ethanol on I_Gly_, we compared the peak amplitudes of currents induced by different concentrations (10-1000 μM) of glycine in the absence and presence of 43.2 mM ethanol (Fig. 4A). On average, 43.2 mM ethanol potentiated the peak I_Gly_ activated by 30 and 100 μM glycine to 148 ± 14% (*n*=15, p<0.05) and 123 ± 9% (*n* =14, p<0.05) of control, respectively. Conversely, this concentration of ethanol has no significant effect on current induced by glycine at 10 and ≥ 300 μM. Fig.4B presents the glycine concentration-response curves for data obtained from neurons in control solution and in the presence of 43.2 mM ethanol. Ethanol (43.2 mM) shifted the concentration response curve of glycine to the left. The EC_50_ and r^2^ were 80.8± 3.6 μM and 0.99, respectively, in the absence of ethanol and 63.8±3.3 μM and 0.99 in the presence of 43.2 mM ethanol.

## DISCUSSION

### Summary of Results

By using the patch clamp technique and the pharmacological approaches, we show here, for the first time, the existence of both functional GABA_A_Rs and GlyRs in the LHb neurons of young rats. More importantly, these receptors are sensitive to ethanol at pharmacologically relevant concentrations.

### Comparison to Previous Studies

GABA_A_Rs have long been implicated in mediating at least some of the pharmacological actions of ethanol. Acute ethanol administration potentiates GABA-mediated inhibition in many brain areas (for review see Mihic [1]. The effect of acute ethanol on GABA_A_Rs in the LHb has not been investigated before. In this study, ethanol at pharmacologically relevant concentrations reversibly and concentration dependently suppressed I_GABA_ induced by a wide range of concentrations of GABA. Although the underlying mechanism warrants further investigation, this finding may significantly contribute to our understanding of mechanisms of alcohol addiction. For example, it may contribute to the stimulating effect of acute ethanol on the firing of LHb neurons (Ye JH, Zuo W, Li J, Xie G. Mechanisms of regulation of ethanol intake by lateral habenula. Neuroscience meeting abstract, New Orleans Nov, 2012).

As mentioned, recent evidence indicates that GlyRs are widely distributed throughout the mammalian CNS. Here, we show that functional GlyRs exist in the LHb neurons of 10-20 day-old rats. Interestingly, when strychnine and glycine were applied to the neurons at the same time, strychnine inhibits peak I_Gly_ of the LHb neurons with almost the same IC_50_ (220 nM) as that for neurons in the ventral tegmental area of rats of similar age(184 nM) [7a]. Intriguingly, there is about 50% of the I_Gly_ in the LHb neurons is resistant to the high picrotoxin concentrations (>300 μM), indicating that they are mediated by the αβ heteromeric GlyRs, which have a much lower sensitivity to picrotoxin [7b, 7c, 21a, 22]. Previous studies have shown that the β subunit of GlyRs is required for receptor clustering [23]; the finding of possible αβ heteromeric GlyRs in LHb raises the possibility that some GlyRs may be synaptically located. However, additional experiments will need to be undertaken to test this hypothesis.

### Effects of ethanol on I_Gly_

Although there are some studies on the effect of ethanol on GlyRs, there is no consensus on this subject. Some studies have shown that ethanol increases the amplitude of I_Gly_ [15b, 24], and others have found that ethanol (30-40 mM) does not affect the I_Gly_ in immature cultured spinal neurons [25] and in neonate hypoglossal motoneurons (P1-3) [26]. Previously published work from our lab also has shown that ethanol potentiates, depresses, or has no effect on I_Gly_ in neurons freshly isolated from the ventral tegmental area [16, 27]. Several factors may account for such differences such as subunit composition of the GlyRs, phosphorylation state, and types of cells. It has been proposed that α2-containing GlyRs are less sensitive to ethanol than α1-containing GlyRs [13a, 24b, 26]. The present study shows that ethanol at 43.2 mM significantly potentiated the current elicited by 30 μM and by 100 μM glycine. These data indicate that current induced by higher concentrations of glycine is less sensitive to ethanol than those induced by lower concentrations of glycine, which is consistent with our previous report [16]. Interestingly, ethanol’s effect on I_Gly_ of LHb induced by 10 μM glycine was not significant. This may be due to the fact that no appreciable current was induced by10 μM glycine in many of the LHb neurons tested. It is unclear at this time why the effects of ethanol on GABA_A_Rs and GlyRs are different. Whatever the reason is, this finding is intriguing given that these two receptors may have different function on the excitability of LHb neurons.

## CONCLUSION

The neurobiological mechanisms underlying the addictive property of ethanol remain obscure. It is generally accepted that the addictive property of ethanol is linked to its ability to increase the activity of dopaminergic neurons in the ventral tegmental area in the brain. These neurons are under the powerful control of synaptic inputs. Thus, the synaptic regulation of dopaminergic neurons is a key initial step in reward mechanisms leading to alcohol addiction. The majority of the afferents to dopaminergic neurons are GABAergic and usually inhibitory. As mentioned, the LHb has attracted a great deal of attention recently due to its role in the regulation of midbrain dopaminergic system. In summary, the results from this study indicated that functional GABA_A_Rs and GlyRs exist in the somatodendritic membrane of LHb neurons of young rats, and more importantly, these receptors are sensitive to ethanol. These effects of ethanol might be important in ethanol addiction as they may regulate the activity of LHb neurons and their outputs indirectly/directly to the midbrain dopamine neurons.

## LIST OF ABBREVIATIONS

CNS: central nervous system
EtOH: ethanol
GABA_A_R: γ-aminobutyric acid A receptor
GlyR: glycine receptor
I_GABA_: GABAA current
I_Gly_: glycine current
LHb: lateral habenula
